# Research progress on the pathogenesis of AKI complicated by ECMO

**DOI:** 10.1007/s10157-024-02559-7

**Published:** 2024-09-28

**Authors:** Keke Sun, Congcong Yao, Guowu Xu, Jinxiang Wang, Songtao Shou, Heng Jin

**Affiliations:** Department of Emergency Medicine, Tianjin Medical University General Hospital, Tianjin, China

**Keywords:** Acute kidney injury, Extracorporeal membrane oxygenation, Inflammation, Renal hypoperfusion, Ischemia–reperfusion injury

## Abstract

**Background:**

Extracorporeal membrane oxygenation (ECMO) stands as a pivotal intervention for patients grappling with cardiopulmonary insufficiency. However, alongside its therapeutic benefits, ECMO carries the risk of complications, with acute kidney injury (AKI) emerging as a significant concern. The precise pathophysiological underpinnings of AKI in the context of ECMO remain incompletely elucidated.

**Methods:**

A comprehensive literature review was conducted to explore the epidemiology and pathophysiological mechanisms underlying the utilization of ECMO in the management of AKI.

**Results:**

ECMO initiates a multifaceted cascade of inflammatory reactions, encompassing complement activation, endothelial dysfunction, white blood cell activation, and cytokine release. Furthermore, factors such as renal hypoperfusion, ischemia–reperfusion injury, hemolysis, and fluid overload exacerbate AKI. Specifically, veno-arterial ECMO (VA-ECMO) may directly induce renal hypoperfusion, whereas veno-venous ECMO (VV-ECMO) predominantly impacts pulmonary function, indirectly influencing renal function.

**Conclusion:**

While ECMO offers significant therapeutic advantages, AKI persists as a potentially fatal complication. A thorough comprehension of the pathogenesis underlying ECMO-associated AKI is imperative for effective prevention and management strategies. Moreover, additional research is warranted to delineate the incidence of AKI secondary to ECMO and to refine clinical approaches accordingly.

## Definition of extracorporeal membrane oxygenation (ECMO)

**Fig. 1:**
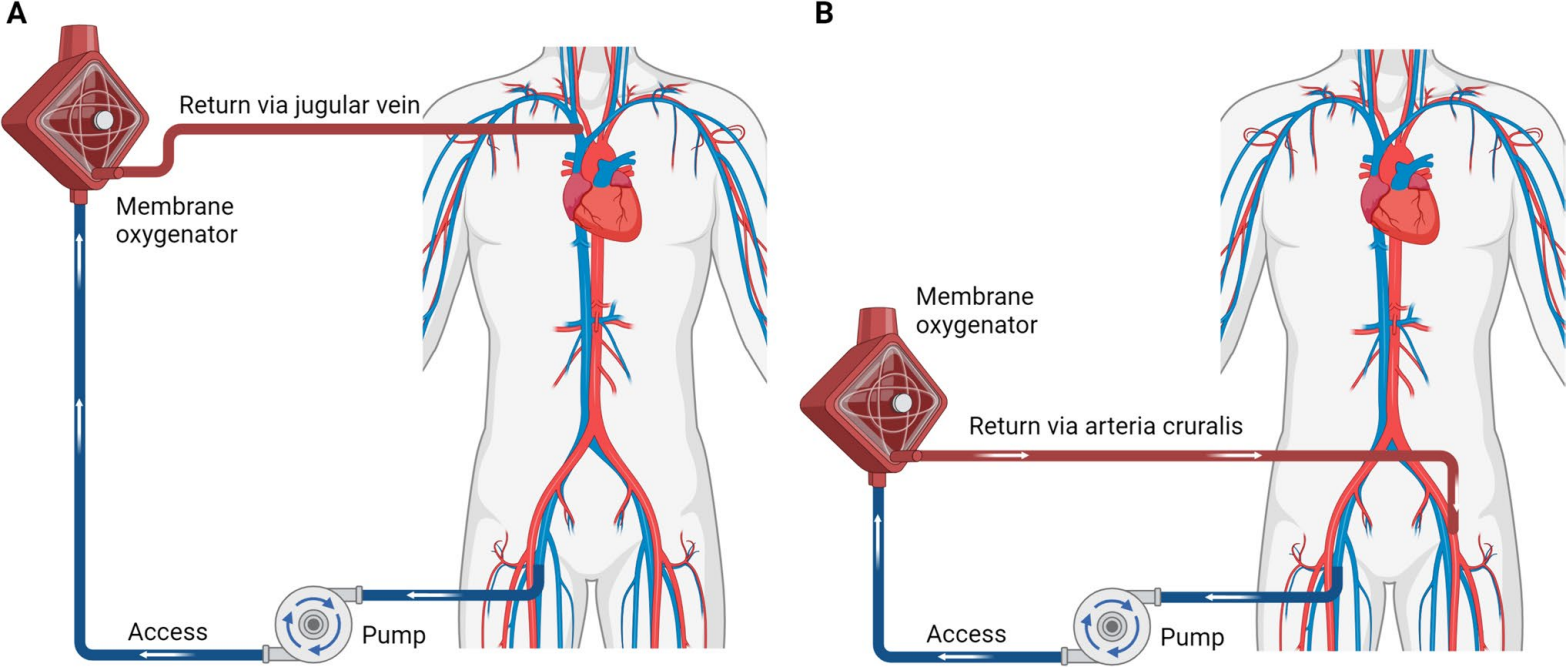
**A** VV-ECMO: right femoral vein to right internal jugular vein; **B** VA-ECMO: right femoral vein to left femoral artery

ECMO stands as a pivotal life-sustaining therapy aimed at facilitating oxygenation and eliminating carbon dioxide to supplement or substitute compromised cardiopulmonary function. This technology pumps the patient’s blood out of the body, followed by its oxygenation and carbon dioxide elimination within specialized apparatuses housing an oxygenator and carbon dioxide scavenger. Subsequently, the oxygen-saturated blood is returned to the patient’s circulation, thereby ensuring the maintenance of adequate oxygenation and ventilation throughout the body [1]. According to the different routes of blood return, ECMO can be categorized into two main types, namely, veno-arterial ECMO (VA-ECMO), veno-venous ECMO (VV-ECMO) (Fig. 1).

### VV-ECMO

VV-ECMO involves the extraction of blood from a vein, typically the jugular or femoral vein. This blood is then routed through an oxygenator for oxygenation before being reintroduced into the patient’s venous system [2]. Common access points for blood retrieval include femoral vein cannulation, internal jugular vein catheterization, or bilateral femoral vein catheterization. The blood follows a path from the veins to the ECMO system, then back to the veins, entering the right atrium, right ventricle, pulmonary artery, lungs, pulmonary vein, left atrium, left ventricle, and finally, the aorta, completing the circulatory loop. VV-ECMO operates in series with the native pulmonary circulation, enriching the blood with oxygen before it re-enters the lungs. Consequently, the lungs are relieved of some of their oxygenation responsibilities, affording them a period of rest. It is essential to consider the status of cardiac function when opting for VV-ECMO therapy. In cases of cardiac failure leading to circulatory collapse, the heart’s ability to maintain circulation is compromised, potentially resulting in fatal outcomes. Therefore, prior to initiating VV-ECMO support, an evaluation of cardiac function is imperative, ensuring that ECMO therapy is administered with due regard to maintaining adequate cardiac function.

### VA-ECMO

VA-ECMO is a medical intervention utilized to provide cardiovascular support by extracting blood from a patient’s vein and subsequently returning oxygenated blood to the patient’s arteries [3]. Typically, blood is withdrawn from the femoral vein, while the femoral artery serves as the conduit for re-oxygenated blood delivery. Nonetheless, due to the technical challenges associated with the femoral vasculature, alternative approaches may be warranted, such as cervical vessel catheterization or direct cardiac vessel access in neonates. The circulatory path involves blood transitioning from veins to the ECMO circuit, then into the arterial system, facilitating systemic circulation before ultimately returning to the venous system. This process effectively supplements cardiac and pulmonary function, making VA-ECMO a viable option for patients experiencing lung injury, respiratory failure concomitant with cardiac insufficiency, or cardiac arrest. However, prolonged employment of VA-ECMO heightens the risk of intracardiac and intrapulmonary thrombosis due to stagnant blood flow within these organs.

## Epidemiology

Acute kidney injury (AKI) stands as a prevalent complication among patients undergoing ECMO. In a comprehensive study involving adult patients, AKI manifested in 44.9% of 10,282 ECMO-treated individuals. The elevated incidence of AKI in patients undergoing ECMO has been attributed to multiple etiological factors. These include hemodynamic instability stemming from cardiac or respiratory insufficiency and circuit-related issues. Specifically, the constant, no pulsatile flow associated with VA-ECMO and the mechanical stress causing intravascular hemolysis are highlighted as influential factors [4–7]. Notably, the in-hospital mortality rate surged by 3.7-fold among patients necessitating renal replacement therapy (RRT) due to AKI during ECMO therapy [8]. Similarly, investigations focusing on pediatric cohorts delineate a combined AKI incidence of 61.9%, with 40.9% necessitating RRT. Comparative analysis underscores a substantial elevation in in-hospital mortality, with patients encountering AKI and those requiring RRT during ECMO exhibiting 1.70 and 3.64 times higher mortality rates, respectively, in contrast to their non-AKI counterparts [9]. Explorations into AKI etiology within ECMO cohorts underscore age as a pivotal determinant, with advanced age predisposing individuals to heightened AKI susceptibility. Moreover, preexisting conditions such as diabetes, hypertension, and chronic kidney disease exacerbate AKI vulnerability. Furthermore, the duration of ECMO support correlates positively with AKI incidence, with prolonged treatment durations escalating risk levels. In addition, the severity of the patient’s underlying pathophysiology, including shock and severe infections, significantly influences AKI development [10, 11].

However, within the cohort, VA-ECMO recipients experienced graver outcomes in terms of AKI occurrences. Mortality rates associated with AKI and severe AKI during VA-ECMO treatment reached 60% and 83%, respectively. Notably, the risk of death for severe AKI was 1.94 times greater than that for non-severe AKI cases within this group. Conversely, VV-ECMO patients exhibited lower mortality rates for AKI (44.4%) and severe AKI (55.8%), albeit with a similar trend of increased risk of death associated with severe AKI compared to non-severe AKI, albeit slightly lower at 1.79 times [12]. Existing literature predominantly supports the notion that VA-ECMO recipients exhibit a higher prevalence of AKI over prolonged observation periods compared to their VV-ECMO counterparts [11, 13, 14].

VA-ECMO serves as a modality for circulatory support in the presence or absence of respiratory insufficiency, providing oxygenation while supplementing cardiac output. Consequently, cardiac output in VA-ECMO patients comprises a blend of non-pulsatile arterial flow from the ECMO circuit and pulsatile arterial blood from the native cardiac output. In contrast, VV-ECMO is employed primarily in cases of profound or refractory respiratory failure. It facilitates oxygenation and CO_2_ removal while preserving the natural pulsatility of cardiac output, potentially mitigating hemodynamic perturbations and preserving renal perfusion. The pulsatile nature of blood flow in VV-ECMO may confer advantageous effects on renal microcirculation and perfusion compared to the non-pulsatile flow characteristic of VA-ECMO. Consequently, the favorable outcomes associated with VV-ECMO could be attributed to prompt correction of blood gas derangements, augmentation of systemic oxygenation, and reduction of oxygen consumption. On the other hand, VA-ECMO serves to restore adequate end-organ perfusion in conditions such as myocardial infarction, end-stage or refractory heart failure, decompensated heart failure, and cardiogenic shock, which are recognized triggers for AKI. Moreover, conditions such as pulmonary hypertension and elevated right atrial pressure are established risk factors for exacerbating renal dysfunction in heart failure patients. In cases of severe heart failure necessitating VA-ECMO support, a diminished ejection fraction can precipitate left ventricular dilation and exacerbate right-sided heart failure, culminating in venous congestion and subsequent AKI. Given the inherent disparity between VA and VV-ECMO, AKI in VA-ECMO patients tends to manifest in a more severe clinical phenotype.

## Physiopathologic mechanism

### Inflammation/immune response

#### Complement system

**Fig. 2:**
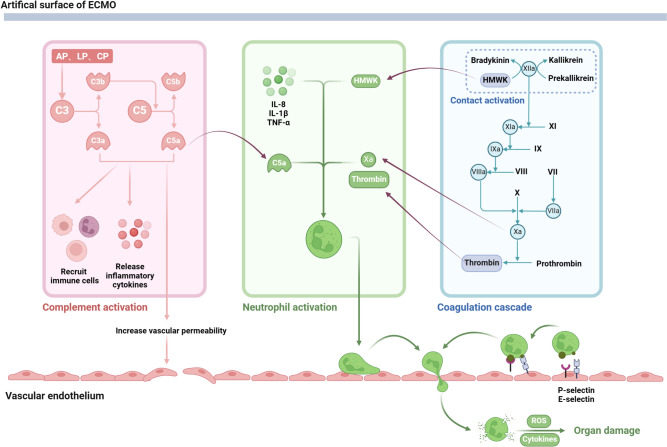
Inflammatory response to ECMO. During ECMO, the complement and contact systems are activated due to blood–biomaterial interactions. The replacement complement pathway is primarily responsible for the production of allergic toxins C3a and C5a. The contact system is responsible for the production of the activation factor XII, which induces the endogenous clotting pathway leading to the formation of thrombin. The products produced by these systems promote the production of pro-inflammatory cytokines and have a direct effect on white blood cells, platelets, and vascular endothelium. In particular, neutrophils are activated, leading to increased neutrophilic infiltration in the tissues, ultimately leading to organ damage. AP, alternative pathway; LP, lectin pathway; CP, classical pathway; HMWK, high-molecular-weight kininogen

Upon initiation of ECMO, the complement system swiftly emerges as one of the foremost host defense mechanisms engaged by the human body. Among the three activation pathways (classical pathway, alternative pathway, and lectin pathway), the alternative pathway holds particular significance in the context of ECMO. Notably, the alternative pathway can be directly activated by blood and biomaterial within the ECMO circuit, leading to the cleavage of C3. Consequently, it is widely postulated that the alternative pathway serves as the principal mechanism driving complement activation during ECMO [15]. C3 and C5 undergo cleavage, yielding C3a, C3b, C5a, and C5b, which are all potent allergens. These substances amplify WBC recruitment, heighten vascular permeability, induce smooth muscle contraction, and stimulate the release of additional inflammatory mediators. Nonetheless, advancements in circuit and pump technologies, coupled with the adoption of modern heparin-binding circuits, have facilitated a reduction in complement activation [16]. Limited research, primarily dating back to the 1990s, has identified complement activation in various settings including in vitro ECMO models, neonatal ECMO, and adult ECMO [17–20]. In recent years, investigations have examined serum samples collected from patients undergoing VA-ECMO on both day 1 and day 3 following initiation. These studies have revealed differential expression of complement C3 and complement factor D compared to control groups [21]. Furthermore, in vitro experiments have demonstrated that the administration of the anticomplement agent APT070 markedly attenuates complement and neutrophil activation, thereby mitigating the inflammatory consequences associated with cardiopulmonary bypass (CPB) [22] (Fig. 2).

#### Endothelial cells

Although vascular endothelial cells do not come into direct contact with the ECMO circuit, they serve as pivotal contributors to the immune-inflammatory response. Endothelial dysfunction stands as a significant prognostic indicator of adverse outcomes among critically ill patients undergoing ECMO therapy. Within the ECMO system, circulating blood generates inflammatory mediators that broadly activate endothelial cells, prompting the secretion of pro-inflammatory cytokines and upregulation of adhesion molecules. Consequently, this cascade fosters the migration of WBC. Central to this process are factors such as TNF-α, IL-1β, complement products, and reactive oxygen species (ROS). Subsequently, activated neutrophils are recruited, adhere to endothelial surfaces, and migrate, culminating in ECMO-related end-organ injury.

#### White blood cell

Some scholars posit that ECMO serves as a “second hit” in leukocyte pathophysiology among critically ill patients, with the activation of neutrophils representing a pivotal event in this cascade [23]. Neutrophils can be stimulated by various factors (e.g., FXa, thrombin in the coagulation system, HMWK in the contact system, C5a, TNF-α in the immune system), leading to recruitment of neutrophils and adhesion to the endothelium. Upon activation, neutrophil degranulation ensues, resulting in the release of an array of cytotoxic enzymes such as neutrophil elastase, myeloperoxidase, and lysozyme. Moreover, neutrophils can generate cytotoxic reactive oxygen species via the “respiratory burst” mechanism and migrate to tissues through the bloodstream, thereby instigating organ damage during ECMO support. Furthermore, monocytes undergo activation in response to ECMO, albeit at a slower pace than neutrophils, and subsequently secrete a diverse array of pro-inflammatory cytokines.

#### Cytokines

During ECMO, the body’s immune response ensues, giving rise to the production of both pro-inflammatory and anti-inflammatory factors. When the equilibrium between these factors is disrupted, it precipitates severe inflammatory responses and subsequent organ dysfunction. Below, we delineate the cytokines most extensively investigated in the context of ECMO.

Tumor necrosis factor-alpha (TNF-α), primarily synthesized by activated mononuclear macrophages, stands as a prominent pro-inflammatory factor. Its actions include the activation of neutrophils, induction of immune responses in endothelial cells, and augmentation of thrombin formation. In a study encompassing 42 adult ECMO-treated patients, TNF-α levels within the survival cohort exhibited a decline 24 h post-ECMO initiation, notably registering significantly lower levels compared to the non-survival group prior to withdrawal. Furthermore, in porcine ECMO models inducing systemic inflammatory response syndrome (SIRS), intestinal mucosal mast cells have been implicated in the rapid surge of TNF-α immediately following ECMO commencement [24]. Among neonates undergoing ECMO, non-survivors exhibit an elevated ratio of TNF-α to its receptor antagonist, a trend correlating with adverse outcomes [25].

Interleukin-6 (IL-6) is a multifaceted cytokine with dual pro-inflammatory and anti-inflammatory properties. It orchestrates the induction and activation of T cells, facilitates B cell differentiation, downregulates the expression of other pro-inflammatory factors, and upregulates the expression of anti-inflammatory factors. In a prospective study spanning two centers and involving 99 neonatal and pediatric ECMO patients, peak IL-6 levels were notably elevated 1 day post-ECMO initiation among children who succumbed compared to those who survived until hospital discharge [26]. Elevated IL-6 levels have been identified as an independent predictor of in-hospital mortality in COVID-19 patients receiving ECMO therapy [27]. A collaborative investigation by Risnes et al. revealed discernible differences in IL-6 levels between survivors and non-survivors, with IL-6 levels rapidly normalizing within 2 days among survivors, whereas persistently elevated levels were observed until demise in non-survivors [28]. Hence, early assessment of IL-6 levels following 2–3 days of ECMO support may serve as a prognostic indicator for clinical outcomes.

Interleukin-8 (IL-8) serves as a primary pro-inflammatory factor known for its ability to activate neutrophils and act as a chemoattractant for neutrophils, basophils, and T cells. Research has indicated elevated levels of IL-8 following ECMO initiation [29–31]. Interestingly, the temporal pattern of IL-8 concentration mirrors that of TNF-α, with an elevation observed within the initial 15 min of ECMO commencement. However, despite its association with ECMO, IL-8 has not been established as a reliable marker for clinical outcomes in ECMO patients.

Interleukin-10 (IL-10), primarily sourced from mononuclear macrophages and T helper cells, functions as an anti-inflammatory factor. In a clinical observational study, IL-10 levels exhibited an increase in patients undergoing VA-ECMO prior to the onset of AKI, followed by a subsequent decline within 48 h post-commencement of circulatory support [32]. Given its elevation preceding changes in creatinine levels, IL-10 holds promise as an early biomarker for AKI onset in ECMO patients. However, the efficacy of early intervention guided by IL-10 elevation in improving outcomes remains to be elucidated.

#### Coagulation cascade and inflammatory response interact

The contact system comprises several plasma proteins, including factor XII, factor XI, high-molecular-weight kininogen (HMWK), and prekallikrein (PK). Upon contact with external surfaces such as tubing, the intrinsic coagulation pathway is triggered, leading to the cleavage of factor XII into factors XIIa and XIIf. Factor XIIa subsequently converts PK into activated kallikrein and HMWK into bradykinin. Kallikreins, in turn, activate neutrophils, while bradykinin stimulates the release of nitric oxide (NO), TNF-α, and IL-10. In the context of ECMO, exogenous clotting pathways typically exert minimal influence. Activation of the contact system ultimately triggers the activation of the endogenous clotting pathway [33]. However, regardless of the pathway involved, the activated factor X within the common pathway catalyzes the conversion of prothrombin into thrombin. Thrombin, in turn, cleaves fibrinogen into fibrin, precipitating blood clot formation. Investigations have demonstrated that assessments of prothrombin time, activated partial thromboplastin time, thrombin time, and clotting factor levels collectively indicate the activation of both endogenous and exogenous clotting pathways during ECMO [33, 34].

#### Platelet

Upon encountering the surface of the ECMO system, platelet pseudopods recognize non-biological materials, thereby initiating platelet activation [35]. Furthermore, platelets can be activated by thrombin and complement activators. Once activated, platelets circulate within the bloodstream, adhere to fibrin, undergo morphological changes, and release granular contents comprising chemokines, pro-inflammatory factors, proteases, adhesion factors, growth factors, angiogenic factors, and hemostatic factors. Concurrently, upregulation of P-selectin on the platelet surface facilitates binding with leukocytes, inducing leukocytes to secrete pro-inflammatory cytokines and prompting monocytes to express tissue factors. This cascade further amplifies the inflammatory response of platelets, WBC, and endothelial cells to ECMO, exacerbating SIRS [36]. The intricate interplay between platelet function and the body’s immune and inflammatory responses poses a complex mechanism with uncertain implications on the body, thus constituting a key challenge to be addressed.

### Renal hypoperfusion

**Fig. 3:**
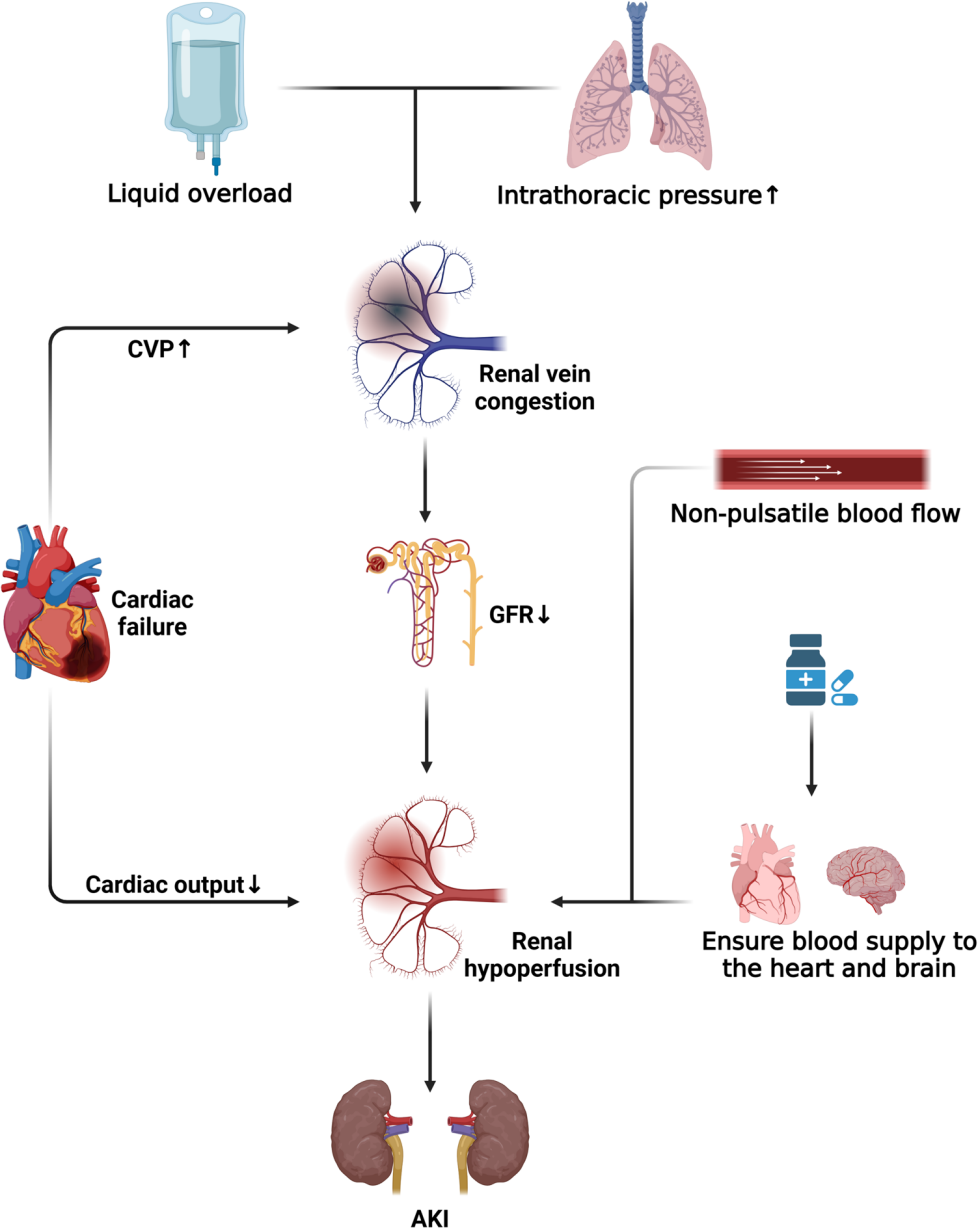
Renal hypoperfusion in ECMO. CVP, central venous pressure; GFR, glomerular filtration rate; AKI, acute kidney injury

Before initiating ECMO, most patients receive relevant treatments (such as massive intravenous fluid infusion, diuretics, nephrotoxic drugs, etc.) aimed at maintaining hemodynamic stability and ensuring adequate perfusion of vital organs like the heart, lungs, and brain. However, these interventions may not be conducive to renal function. In patients with heart failure, decreased cardiac output can result in renal hypoperfusion. Untreated, prerenal AKI may progress to renal AKI, potentially culminating in renal cortical necrosis [37]. Moreover, heart failure often leads to increased central venous pressure and renal venous pressure, consequently reducing the glomerular filtration rate. Furthermore, treatments such as mechanical ventilation and aggressive fluid resuscitation can exacerbate renal hypoperfusion. Positive end-expiratory pressure (PEEP) during ECMO can elevate intrathoracic pressure, diminish venous return, reduce cardiac output, and increase ventricular afterload, thereby elevating systemic venous pressure and venous congestion. This can lead to renal hypoperfusion, impair renal excretory function, and exacerbate AKI progression [38]. During ECMO, low blood volume secondary to hypotension may necessitate large-volume fluid therapy to improve hemodynamics. However, fluid therapy poses a dual risk, while it is administered to prevent AKI, excessive fluid administration itself can induce AKI. Fluid overload can compromise tissue oxygenation and pulmonary oxygen transport, ultimately resulting in dysfunction of the heart, brain, lungs, and other organs, as well as increased renal interstitial pressure. The kidney’s ability to withstand increased stress is limited due to the constraint of the renal capsule, leading to congestion, diminished renal perfusion, reduced glomerular filtration, and aggravated AKI [39]. In addition, mechanical blood flow during ECMO is non-pulsatile, which can detrimentally affect renal cortical blood flow, upregulate the renin–angiotensin–aldosterone system (RAAS), induce systemic vasoconstriction, diminish renal blood flow, promote renal hypoperfusion, and exacerbate AKI [40] (Fig. 3).

### Ischemia–reperfusion injury

**Fig. 4:**
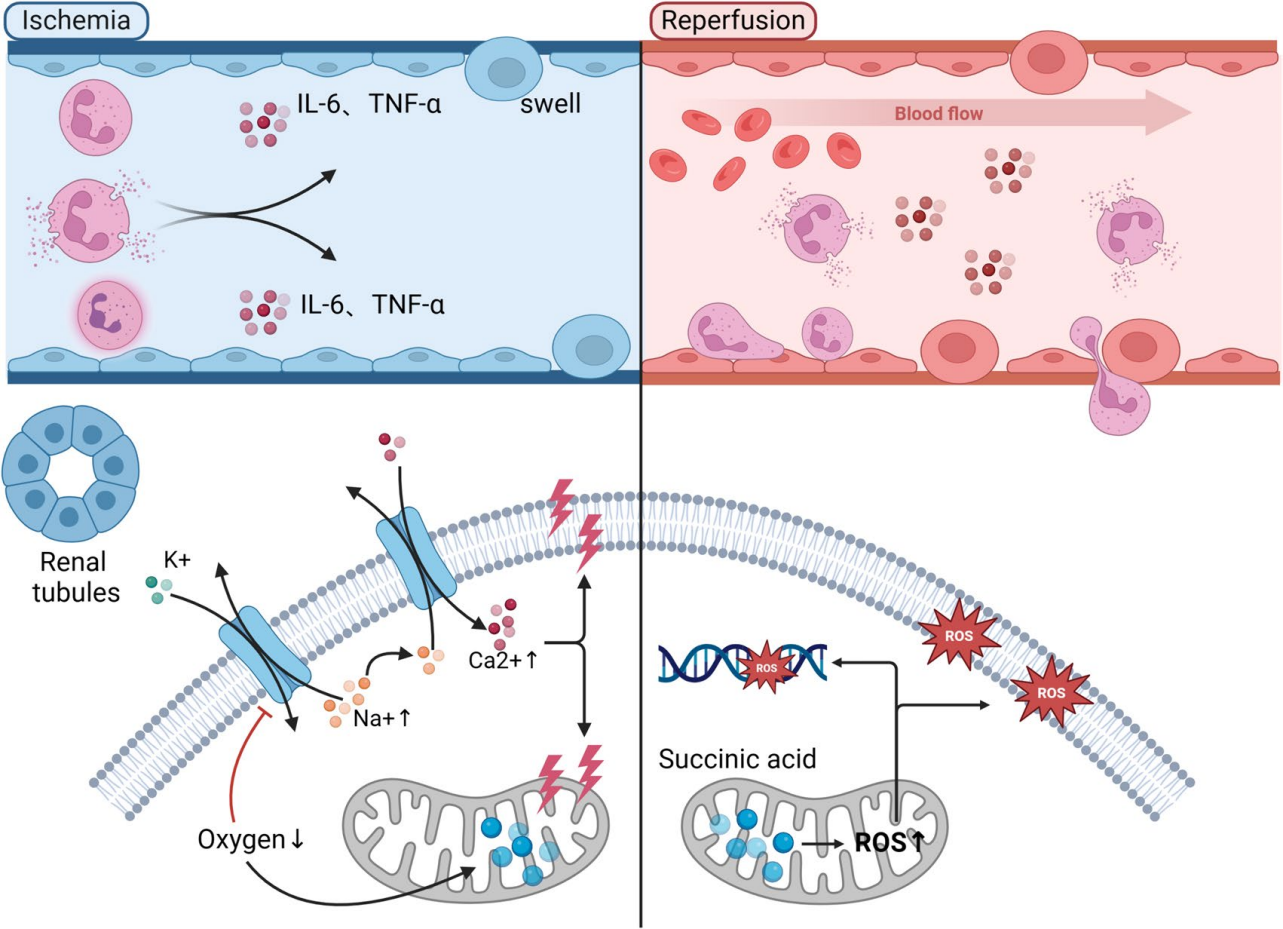
Ischemia–reperfusion injury during ECMO

Ischemia–reperfusion injury stands as the primary cause of AKI, characterized by a complex pathophysiology that remains incompletely understood. Key factors contributing to this process include free radicals, calcium overload, and inflammation. The ECMO-assisted treatment process can be delineated into two stages. The initial stage, preceding ECMO initiation, encompasses a series of interventions (such as fluid therapy, diuretics, vasoconstrictors, etc.) often detrimental to renal function, culminating in renal hypoperfusion—the ischemic phase—marking the onset of kidney injury. Granulocytes serve as the primary source of ROS [41]. During ischemia, succinic acid accumulates in mitochondria and undergoes rapid oxidation upon blood reperfusion, yielding significant ROS production [42]. Under normal physiological conditions, intracellular and extracellular calcium ion concentrations are regulated through active calcium pump transport and Na^+^–Ca^2+^ exchange protein. However, during renal hypoperfusion, reduced ATP synthesis and Na^+^–K^+^-ATPase activity impair normal Na^+^–K^+^ exchange, elevating intracellular Na^+^ levels and facilitating Ca^2+^ influx via Na^+^–Ca^2+^ channels. This results in intracellular calcium overload, mitochondrial structural and functional impairment, metabolic pathway dysregulation, and ROS generation. ROS induce cellular damage through protein, lipid peroxidation, and DNA damage, ultimately leading to tubular necrosis [43]. Moreover, ischemia fosters metabolite accumulation and tissue debris deposition, provoking acute inflammation. Renal parenchymal cells and white blood cells release various chemokines and cytokines, instigating inflammation. These inflammatory mediators recruit white blood cells to ischemic tissues, causing endothelial cell damage, swelling, blood flow obstruction, and microcirculatory dysfunction. Following ECMO initiation, patient hemodynamics and oxygenation improve, facilitating restoration of circulating blood flow to hypoxic kidney tissues and cells—the reperfusion phase. However, reperfusion elicits the influx of numerous white blood cells into tissues via the bloodstream, exacerbating the inflammatory response and worsening kidney damage [44] (Fig. 4).

### Hemolytic injuries

During ECMO, blood undergoes continuous exposure to non-physiological turbulence fields and high shear forces as it traverses a cardiopulmonary system comprising mechanical pumps, artificial lungs, microplug filters, ultrafilters, and intubation lines. This exposure can prompt immediate or delayed hemolysis of red blood cells due to rolling, impact, shear, differential pressure, and other injuries [45]. As previously discussed, ECMO can incite systemic inflammatory response syndrome through various adjuvant therapy modalities. Notably, the unique double-concave disc-like structure of red blood cells (RBC) results in the largest contact area between the RBC membrane and plasma among human cells, rendering RBC particularly susceptible to damage from inflammatory mediators. Hemoglobin (Hb) release from damaged or stretched cells results in excess free Hb (FHb), an endogenous toxin capable of inducing acute tubular necrosis through multiple pathways. First, FHb can induce oxidative stress in the kidneys, primarily mediated by reactive nitrogen and oxygen species (RNOS), the main oxidants in the human body. Under normal circumstances, RNOS play crucial roles in cell signaling, immunity, cell defense, and microvascular function [46]. However, hemolysis during ECMO can elevate levels of FHb and free iron components, thereby disturbing this delicate balance and inflicting damage upon tubular epithelial cells [47]. Moreover, FHb may undergo conversion to methemoglobin, which subsequently precipitates within renal tubules, forming tubular obstructions [48]. Furthermore, FHb can bind to endothelial relaxation factor NO to form nitroso-hemoglobin, whose inactivation results in renal vasoconstriction [49]. Ultimately, hemolysis during ECMO precipitates systemic imbalances, leading to renal cell and substructure damage, impaired renal function, and subsequent AKI.

## Risk factors of AKI in ECMO patients

While ECMO was initially designed to serve as an emergency treatment for neonates facing respiratory failure, its utilization in adult patients has notably expanded, resulting in a current prevalence that surpasses the aggregate count of pediatric and neonatal recipients [50–54]. Moreover, patients who undergo ECMO placement are among the most critically ill and, consequently, are at an elevated risk for developing AKI before cannulation [55]. This risk is attributed to the severity of their underlying conditions, the etiology of their primary disease, and the treatments they receive, which may include respiratory failure, cardiac failure, hypotension necessitating vasopressor support, cardiac arrest, ischemia, and exposure to nephrotoxic agents [56, 57]. A population-based meta-analysis has identified a correlation between AKI and several factors in adult ECMO patients. Severe AKI is more strongly associated with higher Sequential Organ Failure Assessment (SOFA) scores, the presence of diabetes mellitus, and longer durations of ECMO support [11]. A retrospective cohort study conducted on patients who received ECMO support found that the severity of underlying disease, cardiac dysfunction prior to ECMO initiation, and blood lactate levels at 24 h post-ECMO initiation were independent risk factors significantly associated with the development of AKI [14]. However, the early detection facilitated by some biomarkers could support early risk stratification based on evidence, aiding in the diagnostic process, AKI monitoring, and the timely initiation or discontinuation of “renal protective” interventions for ECMO patients [58]. The most research has been conducted on neutrophil gelatinase-associated lipocalin (NGAL) and cystatin C (CysC) for their diagnostic potential in AKI [59]. Other biomarkers with potential include kidney injury molecule-1 (KIM-1), liver-type fatty acid-binding protein (L-FABP), insulin-like growth factor-binding protein 7 (IGFBP7), and tissue inhibitor of metalloproteinases-2 (TIMP-2) [60].

## Delivery of continuous renal replacement therapy (CRRT) to ECMO patients

In the context of ECMO, the need for substantial fluid therapy is common to enhance hemodynamics, often due to hypotension that may stem from hypovolemia or diminished systemic vascular resistance [61]. The kidneys’ ability to handle this substantial fluid load is pivotal; any impairment can lead to a rapid and marked disruption of fluid balance. The accumulation of clinically relevant fluid is a significant sign of renal dysfunction in clinical settings. A survey encompassing 65 ECMO centers internationally indicated that the main reasons for initiating CRRT during ECMO were for the treatment of fluid overload (43%), its prevention (16%), AKI (35%), and correction of electrolyte imbalances (4%) [62]. However, the provision of prolonged ECMO support is linked to a heightened risk for the onset of AKI [62]. As a result, renal replacement therapy, predominantly in the form of CRRT, is employed in 30–60% of ECMO patients afflicted with severe AKI [61, 63–65]. In the context of ECMO, several RRT options are available, such as continuous veno-venous hemofiltration, continuous veno-venous hemodialysis, continuous veno-venous hemodiafiltration, and slow continuous ultrafiltration. The combination of ECMO with CRRT can be executed in multiple ways, each presenting distinct advantages and disadvantages [66]. Despite the variety of methods, no consensus on a standard approach has been reached. The optimal timing for initiating CRRT during ECMO remains a subject of limited research, with no clinical trials specifically dedicated to this question to date. A comprehensive meta-analysis of adult ECMO patients has indicated that early initiation of CRRT may enhance survival rates. Similar findings have been observed in pediatric ECMO patients, where an early start to CRRT is linked to better outcomes. For patients on ECMO and critically ill patients in general, the decision to start CRRT should be made on an individual basis, with daily reassessments that weigh the potential risks and benefits. It is widely recognized that fluid overload (FO) is a significant predictor of higher mortality, particularly among patients with heart and respiratory failure, and should be considered a primary indicator for the proactive initiation of CRRT [61]. It is now recognized that a positive fluid balance is associated with mortality in a manner that is independent, thus emphasizing the importance of its prevention [64]. CRRT can be instrumental in controlling fluid balance with timeliness and accuracy, especially when initiated as part of an early treatment approach [67].

## Conclusion

The utilization of ECMO technology has become integral to the realm of clinical acute and critical care medicine. It has been demonstrated that ECMO support allows for the temporary rest and recovery of cardiac and pulmonary function, a feat not attainable through pharmaceutical interventions or other invasive treatment modalities. Despite advancements in ECMO device refinement and blood protection strategies, AKI persists as a significant complication, significantly impacting patient prognosis. Consequently, investigating the pathogenesis of AKI in the context of ECMO complications holds substantial clinical significance.

This review primarily synthesizes the pathophysiological mechanisms and risk factors associated with AKI in the context of ECMO, as well as the timing for initiating RRT during ECMO. The goal is to provide a theoretical framework for the clinical prevention and management of AKI within the ECMO environment. Furthermore, given the escalating utilization of ECMO, there exists a notable dearth of foundational research data concerning AKI incidence, highlighting an imperative direction for future investigations.
